# (2*E*)-3-(2-Chloro-7-methyl­quinolin-3-yl)-1-(6-chloro-2-methyl-4-phenyl­quinolin-3-yl)prop-2-en-1-one ethanol monosolvate

**DOI:** 10.1107/S1600536813022022

**Published:** 2013-08-14

**Authors:** R. Prasath, S. Sarveswari, Seik Weng Ng, Edward R. T. Tiekink

**Affiliations:** a Department of Chemistry, BITS, Pilani – K. K. Birla Goa Campus, Goa 403 726, India; b Centre for Organic and Medicinal Chemistry, School of Advanced Sciences, VIT University, Vellore 632 014, India; c Department of Chemistry, University of Malaya, 50603 Kuala Lumpur, Malaysia; d Chemistry Department, Faculty of Science, King Abdulaziz University, PO Box 80203 Jeddah, Saudi Arabia

## Abstract

In the title ethanol solvate, C_29_H_20_Cl_2_N_2_O·C_2_H_5_OH, the quinolinyl residues form a dihedral angle of 46.41 (4)° with each other, and each is inclined [C_p_—C—C=O and C=C—C—C_p_ (p = pyridyl) torsion angles = 54.8 (2) and 144.44 (19)°, respectively] with respect to the almost planar bridging prop-2-en-1-one residue [O=C—C=C torsion angle = −4.1 (3)°]. The ethanol solvent mol­ecule is disordered over two positions of equal occupancy and is located close to a centre of inversion. These mol­ecules reside in cavities defined by the organic mol­ecules, which are connected into a three-dimensional architecture by C—H⋯Cl, C—H⋯O and C—H⋯N inter­actions, as well as π–π contacts [inter-centroid distances = 3.5853 (10) and 3.8268 (11) Å], each involving pyridyl rings.

## Related literature

For background details and the biological applications of quinolin­yl/chalcone derivatives, see: Joshi *et al.* (2011 ▶); Prasath *et al.* (2013*a* ▶). For a related structure, see: Prasath *et al.* (2013*b* ▶).
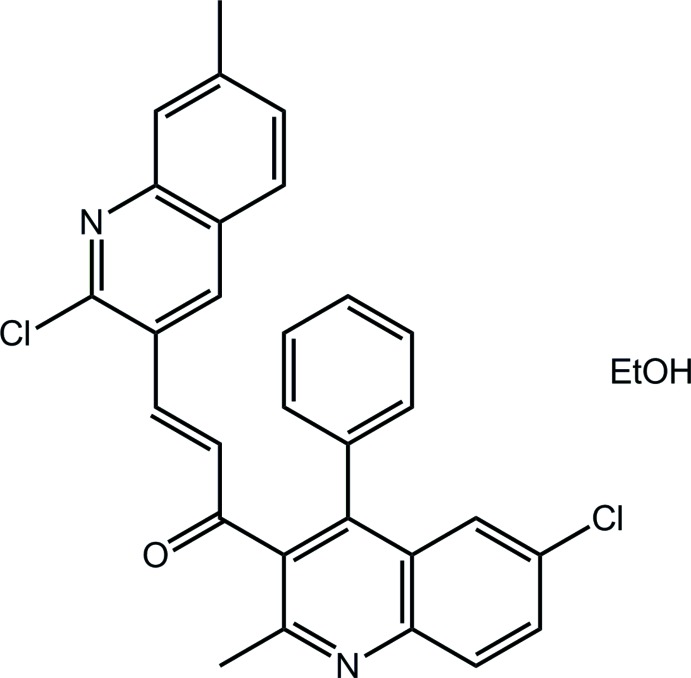


## Experimental

### 

#### Crystal data

C_29_H_20_Cl_2_N_2_O·C_2_H_6_O
*M* *_r_* = 529.44
Triclinic, 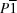

*a* = 9.1621 (3) Å
*b* = 11.3598 (4) Å
*c* = 13.1879 (5) Å
α = 74.017 (3)°
β = 85.995 (3)°
γ = 77.683 (3)°
*V* = 1289.07 (8) Å^3^
*Z* = 2
Cu *K*α radiation
μ = 2.52 mm^−1^
*T* = 100 K
0.40 × 0.30 × 0.20 mm

#### Data collection

Agilent SuperNova Dual diffractometer with an Atlas detector
Absorption correction: multi-scan (*CrysAlis PRO*; Agilent, 2013 ▶) *T*_min_ = 0.724, *T*_max_ = 1.000
9624 measured reflections
5287 independent reflections
4904 reflections with *I* > 2σ(*I*)
*R*_int_ = 0.020

#### Refinement

*R*[*F* ^2^ > 2σ(*F* ^2^)] = 0.044
*wR*(*F* ^2^) = 0.122
*S* = 1.05
5287 reflections
365 parameters
42 restraints
H-atom parameters constrained
Δρ_max_ = 0.52 e Å^−3^
Δρ_min_ = −0.77 e Å^−3^

### 

Data collection: *CrysAlis PRO* (Agilent, 2013 ▶); cell refinement: *CrysAlis PRO*; data reduction: *CrysAlis PRO*; program(s) used to solve structure: *SHELXS97* (Sheldrick, 2008 ▶); program(s) used to refine structure: *SHELXL97* (Sheldrick, 2008 ▶); molecular graphics: *ORTEP-3 for Windows* (Farrugia, 2012 ▶) and *DIAMOND* (Brandenburg, 2006 ▶); software used to prepare material for publication: *publCIF* (Westrip, 2010 ▶).

## Supplementary Material

Crystal structure: contains datablock(s) general, I. DOI: 10.1107/S1600536813022022/mw2114sup1.cif

Structure factors: contains datablock(s) I. DOI: 10.1107/S1600536813022022/mw2114Isup2.hkl

Supplementary material file. DOI: 10.1107/S1600536813022022/mw2114Isup3.cml

Additional supplementary materials:  crystallographic information; 3D view; checkCIF report

## Figures and Tables

**Table 1:** Hydrogen-bond geometry (Å, °)

*D*—H⋯*A*	*D*—H	H⋯*A*	*D*⋯*A*	*D*—H⋯*A*
C15—H15⋯N2^i^	0.95	2.55	3.335 (2)	140
C25—H25⋯O1^ii^	0.95	2.45	3.394 (3)	170
C26—H26⋯Cl1^iii^	0.95	2.75	3.654 (2)	159

Symmetry codes: (i) 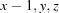; (ii) 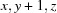; (iii) 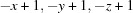.

**Fig. 1.**
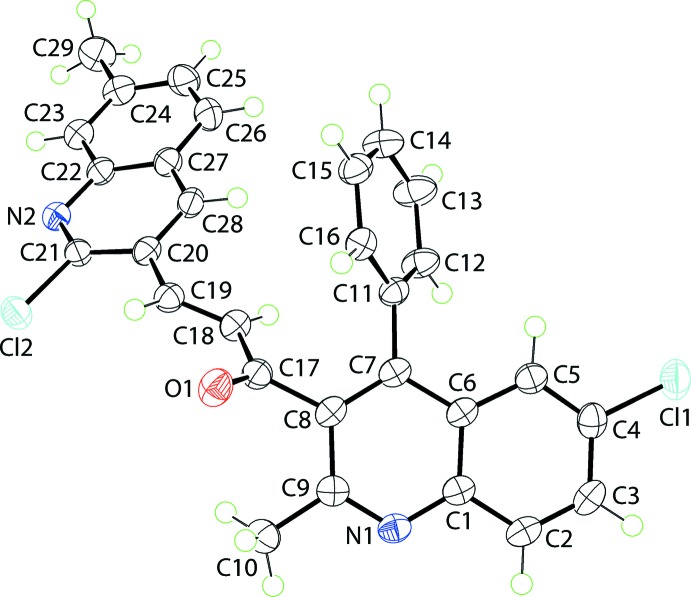
The molecular structure of (I) showing the atom-labelling scheme and displacement ellipsoids at the 70% probability level. The disordered ethanol molecule is not shown.

**Fig. 2.**
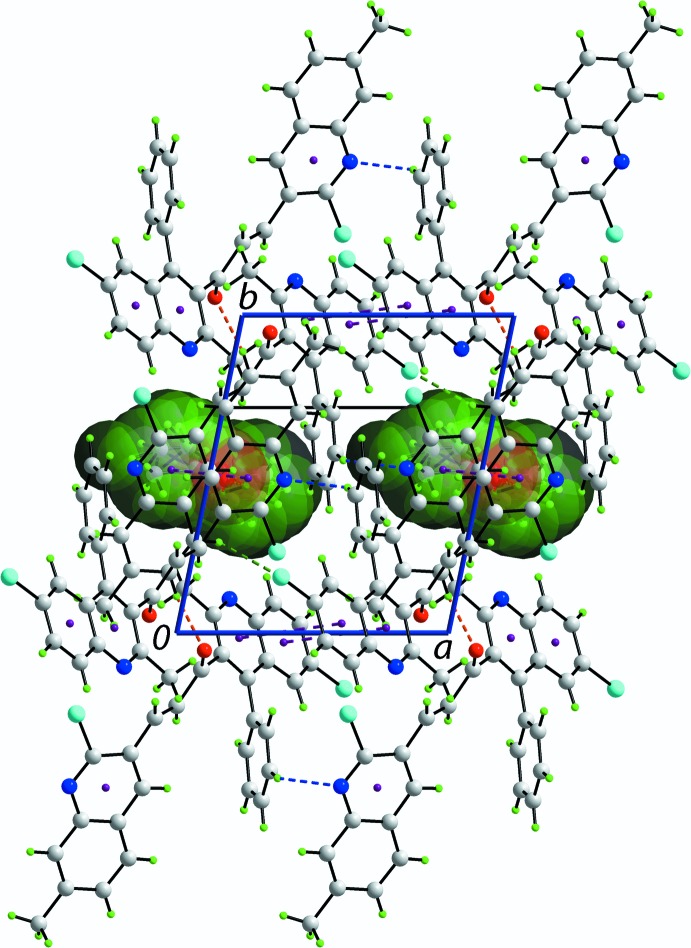
View in projection down the c axis of the unit-cell contents of (I). The disordered ethanol molecules, highlighted in space-filling mode, occupy cavities defined by the organic molecules which are connected by C—H···Cl, C—H···O, C—H···N and π—π interactions, shown as green, orange, blue and purple dashed lines, respectively.

## supplementary crystallographic information

### 1. Comment

Quinoline analogues, including chalcones, have gained much attention due to their bio-activities such as anti-bacterial, anti-fungal, anti-malarial and anti-cancer activities (Joshi *et al.*, 2011; Prasath *et al.*, 2013*a*). It was in this connection that the title compound, (I), was investigated.

The terminal quinolinyl residues in (I), Fig. 1, are inclined to each other forming a dihedral angle of 46.41 (4)°. The bridge between these, *i.e*. the prop-2-en-1-one residue, is planar as seen in the O1—C17—C18—C19 torsion angle of -4.1 (3)°. Each quinolinyl fused ring system is inclined to the central plane: the C9—C8—C17—O1 and C18—C19—C20—C21 torsion angles are 54.8 (2) and 144.44 (19)°, respectively. The phenyl ring is inclined to the pyridyl ring to which it is attached, forming a dihedral angle of 46.28 (9)°. The conformation about the C18═C19 bond [1.337 (3) Å] is *E*.

In a closely related compound, (*2E*)-3-(2-chloro-8-methylquinolin-3-yl)-1-(5,7-dimethylquinolin-6-yl)πrop-2-en-1-one (Prasath *et al.* 2013*b*), the orientation of the N2-quinolinyl residue is to the other side of the molecule to that found in (I); the pyridyl-nitrogen atoms may be considered *syn* in (I).

The quiniolinyl molecules are connected by C—H···Cl, O and N interactions, Table 1, as well as π—π contacts [inter-centroid distances: *Cg*(N2-pyridyl)···*Cg*(N2-pyridyl)^i^ = 3.5853 (10) Å and *Cg*(N1-pyridyl)···*Cg*(C1–C6)^ii^ = 3.8268 (11) Å for symmetry operations *i*: 2 - *x*, 1 - *y*, -*z* and *ii*: 1 - *x*, -*y*, 1 - *z*] to form a three-dimensional architecture. This defines cavities in which residue the highly disordered ethanol molecules, Fig. 2.

### 2. Experimental

A mixture of 3-acetyl-6-chloro-2-methyl-4-phenylquinoline (300 mg, 0.001 *M*) and 2-chloro-7-methylquinoline-3-carbaldehyde (200 mg, 0.001 *M*) in methanol (20 ml) containing potassium hydroxide (0.2 g) was stirred at room temperature for 12 h. Then the reaction mixture was neutralized with dilute acetic acid and the solid that formed was filtered off, washed with distilled ethanol to remove excess of water (from dilute acetic acid), dried and purified by column chromatography using an ethyl acetate-hexane (4:1) mixture to afford compound (I). Re-crystallization was by slow evaporation of its acetone solution, which yielded prisms in 87% yield; *M*.pt: 453–455 K.

### 3. Refinement

Carbon-bound H-atoms were placed in calculated positions [C—H = 0.95–0.98 Å, *U*_iso_(H) = 1.2–1.5*U*_eq_(C)] and were included in the refinement in the riding model approximation. The oxygen-bound H-atoms were treated similarly with O—H = 0.84 Å, and with *U*_iso_(H) = 1.5*U*_eq_(O)]. A disordered ethanol molecule of solvation was found towards the final stages of the refinement. Two positions of half-weight were resolved and these are disordered over a centre of inversion. The 1,2- and 1,3- distances were refined with distance restraints of 1.500 (5) and 2.45 (1) Å, respectively. All atoms were refined with individual anisotropic displacement parameters but these were constrained to be nearly isotropic (ISOR command in *SHELXL97*). Owing to poor agreement, the (0 1 0) reflection was omitted from the final refinement.

### Crystal data

**Table:**

C_29_H_20_Cl_2_N_2_O·C_2_H_6_O	*Z* = 2
*M**_r_* = 529.44	*F*(000) = 552
Triclinic, *P*1	*D*_x_ = 1.364 Mg m^−^^3^
Hall symbol: -P 1	Cu *K*α radiation, λ = 1.54184 Å
*a* = 9.1621 (3) Å	Cell parameters from 5460 reflections
*b* = 11.3598 (4) Å	θ = 3.5–76.2°
*c* = 13.1879 (5) Å	µ = 2.52 mm^−^^1^
α = 74.017 (3)°	*T* = 100 K
β = 85.995 (3)°	Prism, pale-yellow
γ = 77.683 (3)°	0.40 × 0.30 × 0.20 mm
*V* = 1289.07 (8) Å^3^	

### Data collection

**Table:**

Agilent SuperNova Dual diffractometer with an Atlas detector	5287 independent reflections
Radiation source: SuperNova (Cu) X-ray Source	4904 reflections with *I* > 2σ(*I*)
Mirror monochromator	*R*_int_ = 0.020
Detector resolution: 10.4041 pixels mm^-1^	θ_max_ = 76.4°, θ_min_ = 3.5°
ω scan	*h* = −11→11
Absorption correction: multi-scan (*CrysAlis PRO*; Agilent, 2013)	*k* = −14→13
*T*_min_ = 0.724, *T*_max_ = 1.000	*l* = −16→16
9624 measured reflections	

### Refinement

**Table:**

Refinement on *F*^2^	Primary atom site location: structure-invariant direct methods
Least-squares matrix: full	Secondary atom site location: difference Fourier map
*R*[*F*^2^ > 2σ(*F*^2^)] = 0.044	Hydrogen site location: inferred from neighbouring sites
*wR*(*F*^2^) = 0.122	H-atom parameters constrained
*S* = 1.05	*w* = 1/[σ^2^(*F*_o_^2^) + (0.0683*P*)^2^ + 0.7931*P*] where *P* = (*F*_o_^2^ + 2*F*_c_^2^)/3
5287 reflections	(Δ/σ)_max_ < 0.001
365 parameters	Δρ_max_ = 0.52 e Å^−^^3^
42 restraints	Δρ_min_ = −0.77 e Å^−^^3^

### Special details

**Table:**

Geometry. All s.u.'s (except the s.u. in the dihedral angle between two l.s. planes) are estimated using the full covariance matrix. The cell s.u.'s are taken into account individually in the estimation of s.u.'s in distances, angles and torsion angles; correlations between s.u.'s in cell parameters are only used when they are defined by crystal symmetry. An approximate (isotropic) treatment of cell s.u.'s is used for estimating s.u.'s involving l.s. planes.
Refinement. Refinement of *F*^2^ against ALL reflections. The weighted *R*-factor *wR* and goodness of fit *S* are based on *F*^2^, conventional *R*-factors *R* are based on *F*, with *F* set to zero for negative *F*^2^. The threshold expression of *F*^2^ > 2σ(*F*^2^) is used only for calculating *R*-factors(gt) *etc*. and is not relevant to the choice of reflections for refinement. *R*-factors based on *F*^2^ are statistically about twice as large as those based on *F*, and *R*- factors based on ALL data will be even larger.

### Fractional atomic coordinates and isotropic or equivalent isotropic displacement parameters (Å^2^)

**Table:**

	*x*	*y*	*z*	*U*_iso_*/*U*_eq_	Occ. (<1)
Cl1	0.34164 (6)	0.17568 (5)	0.73142 (4)	0.03305 (15)	
Cl2	1.30733 (5)	0.25503 (4)	0.02173 (4)	0.02611 (13)	
O1	0.88501 (16)	0.05550 (13)	0.19850 (11)	0.0273 (3)	
N1	0.83172 (17)	−0.10669 (14)	0.52772 (12)	0.0210 (3)	
N2	1.27879 (17)	0.48253 (14)	0.03288 (12)	0.0200 (3)	
C1	0.7161 (2)	−0.03693 (16)	0.57030 (14)	0.0196 (3)	
C2	0.6659 (2)	−0.09198 (17)	0.67277 (15)	0.0234 (4)	
H2	0.7112	−0.1753	0.7081	0.028*	
C3	0.5532 (2)	−0.02705 (18)	0.72179 (15)	0.0249 (4)	
H3	0.5215	−0.0641	0.7912	0.030*	
C4	0.4849 (2)	0.09544 (18)	0.66766 (15)	0.0232 (4)	
C5	0.5280 (2)	0.15195 (17)	0.56784 (14)	0.0216 (4)	
H5	0.4777	0.2338	0.5325	0.026*	
C6	0.64822 (19)	0.08754 (16)	0.51745 (13)	0.0187 (3)	
C7	0.7056 (2)	0.14158 (16)	0.41531 (13)	0.0186 (3)	
C8	0.8184 (2)	0.06739 (16)	0.37231 (13)	0.0190 (3)	
C9	0.8792 (2)	−0.05784 (16)	0.43182 (14)	0.0197 (3)	
C10	1.0070 (2)	−0.13943 (18)	0.38962 (16)	0.0259 (4)	
H10A	1.0560	−0.2069	0.4482	0.039*	
H10B	1.0792	−0.0891	0.3534	0.039*	
H10C	0.9688	−0.1754	0.3399	0.039*	
C11	0.6480 (2)	0.27469 (16)	0.35914 (14)	0.0196 (3)	
C12	0.6550 (2)	0.36842 (18)	0.40740 (15)	0.0261 (4)	
H12	0.6891	0.3464	0.4779	0.031*	
C13	0.6126 (3)	0.49367 (19)	0.35312 (17)	0.0322 (5)	
H13	0.6185	0.5569	0.3863	0.039*	
C14	0.5613 (2)	0.52672 (18)	0.25008 (16)	0.0296 (4)	
H14	0.5320	0.6123	0.2130	0.036*	
C15	0.5532 (2)	0.43448 (18)	0.20205 (15)	0.0244 (4)	
H15	0.5185	0.4569	0.1317	0.029*	
C16	0.5956 (2)	0.30926 (17)	0.25600 (14)	0.0206 (4)	
H16	0.5888	0.2465	0.2225	0.025*	
C17	0.8820 (2)	0.11464 (17)	0.26361 (14)	0.0206 (4)	
C18	0.9451 (2)	0.22873 (17)	0.24087 (14)	0.0211 (4)	
H18	0.9351	0.2755	0.2915	0.025*	
C19	1.0162 (2)	0.26635 (17)	0.14936 (14)	0.0207 (4)	
H19	1.0267	0.2170	0.1007	0.025*	
C20	1.0786 (2)	0.37970 (17)	0.12036 (14)	0.0199 (3)	
C21	1.2140 (2)	0.38775 (17)	0.06073 (13)	0.0189 (3)	
C22	1.2102 (2)	0.58889 (17)	0.06146 (13)	0.0201 (4)	
C23	1.2797 (2)	0.69349 (18)	0.03304 (14)	0.0230 (4)	
H23	1.3725	0.6884	−0.0041	0.028*	
C24	1.2145 (2)	0.80230 (18)	0.05860 (15)	0.0258 (4)	
C25	1.0754 (2)	0.80895 (19)	0.11348 (16)	0.0292 (4)	
H25	1.0288	0.8846	0.1301	0.035*	
C26	1.0070 (2)	0.70854 (19)	0.14279 (16)	0.0282 (4)	
H26	0.9144	0.7151	0.1800	0.034*	
C27	1.0727 (2)	0.59504 (18)	0.11830 (14)	0.0218 (4)	
C28	1.0093 (2)	0.48750 (17)	0.14737 (14)	0.0216 (4)	
H28	0.9177	0.4893	0.1860	0.026*	
C29	1.2868 (3)	0.9150 (2)	0.02904 (19)	0.0341 (5)	
H29A	1.3926	0.8892	0.0119	0.051*	
H29B	1.2782	0.9524	0.0884	0.051*	
H29C	1.2366	0.9765	−0.0324	0.051*	
O2	0.0422 (5)	0.5120 (4)	0.5307 (3)	0.0565 (10)	0.50
H2O	0.0610	0.4402	0.5726	0.085*	0.50
C30	−0.1004 (7)	0.5851 (5)	0.5640 (6)	0.065 (2)	0.50
H30A	−0.0787	0.6280	0.6155	0.078*	0.50
H30B	−0.1481	0.6495	0.5020	0.078*	0.50
C31	−0.2032 (6)	0.4996 (6)	0.6125 (4)	0.0459 (12)	0.50
H31A	−0.2966	0.5478	0.6335	0.069*	0.50
H31B	−0.1563	0.4371	0.6748	0.069*	0.50
H31C	−0.2244	0.4574	0.5613	0.069*	0.50
O2'	0.0663 (4)	0.4796 (6)	0.6210 (3)	0.0775 (16)	0.50
H2O'	0.1418	0.4618	0.5840	0.116*	0.50
C30'	−0.0577 (5)	0.5156 (6)	0.5604 (4)	0.0400 (11)	0.50
H30C	−0.0743	0.4398	0.5437	0.048*	0.50
H30D	−0.0315	0.5720	0.4929	0.048*	0.50
C31'	−0.2023 (5)	0.5774 (6)	0.5941 (4)	0.0415 (11)	0.50
H31D	−0.2753	0.5959	0.5382	0.062*	0.50
H31E	−0.1922	0.6555	0.6084	0.062*	0.50
H31F	−0.2362	0.5223	0.6583	0.062*	0.50

### Atomic displacement parameters (Å^2^)

**Table:**

	*U*^11^	*U*^22^	*U*^33^	*U*^12^	*U*^13^	*U*^23^
Cl1	0.0348 (3)	0.0304 (3)	0.0363 (3)	−0.0104 (2)	0.0149 (2)	−0.0136 (2)
Cl2	0.0245 (2)	0.0217 (2)	0.0323 (2)	−0.00494 (17)	0.00614 (17)	−0.00892 (18)
O1	0.0370 (8)	0.0258 (7)	0.0227 (6)	−0.0113 (6)	0.0035 (6)	−0.0095 (5)
N1	0.0222 (7)	0.0186 (7)	0.0220 (7)	−0.0057 (6)	−0.0024 (6)	−0.0034 (6)
N2	0.0197 (7)	0.0217 (7)	0.0190 (7)	−0.0052 (6)	−0.0010 (6)	−0.0050 (6)
C1	0.0206 (8)	0.0190 (8)	0.0201 (8)	−0.0069 (7)	−0.0031 (7)	−0.0040 (7)
C2	0.0272 (9)	0.0208 (9)	0.0215 (9)	−0.0087 (7)	−0.0031 (7)	−0.0007 (7)
C3	0.0303 (10)	0.0262 (9)	0.0193 (8)	−0.0135 (8)	0.0024 (7)	−0.0029 (7)
C4	0.0234 (9)	0.0247 (9)	0.0255 (9)	−0.0101 (7)	0.0043 (7)	−0.0103 (7)
C5	0.0233 (9)	0.0191 (8)	0.0234 (9)	−0.0068 (7)	−0.0004 (7)	−0.0053 (7)
C6	0.0209 (8)	0.0187 (8)	0.0182 (8)	−0.0083 (7)	−0.0024 (6)	−0.0039 (6)
C7	0.0213 (8)	0.0178 (8)	0.0186 (8)	−0.0083 (7)	−0.0029 (6)	−0.0040 (6)
C8	0.0213 (8)	0.0201 (8)	0.0174 (8)	−0.0090 (7)	−0.0018 (6)	−0.0040 (7)
C9	0.0195 (8)	0.0195 (8)	0.0219 (8)	−0.0061 (7)	−0.0028 (7)	−0.0062 (7)
C10	0.0238 (9)	0.0257 (9)	0.0274 (9)	−0.0028 (7)	−0.0010 (7)	−0.0075 (8)
C11	0.0205 (8)	0.0183 (8)	0.0196 (8)	−0.0061 (7)	0.0002 (6)	−0.0027 (7)
C12	0.0376 (11)	0.0207 (9)	0.0204 (8)	−0.0065 (8)	−0.0059 (8)	−0.0041 (7)
C13	0.0496 (13)	0.0190 (9)	0.0287 (10)	−0.0068 (8)	−0.0078 (9)	−0.0059 (8)
C14	0.0366 (11)	0.0192 (9)	0.0292 (10)	−0.0032 (8)	−0.0066 (8)	−0.0006 (7)
C15	0.0246 (9)	0.0258 (9)	0.0205 (8)	−0.0049 (7)	−0.0030 (7)	−0.0017 (7)
C16	0.0212 (8)	0.0217 (9)	0.0207 (8)	−0.0077 (7)	0.0001 (7)	−0.0062 (7)
C17	0.0198 (8)	0.0211 (8)	0.0205 (8)	−0.0039 (7)	−0.0005 (7)	−0.0049 (7)
C18	0.0205 (8)	0.0224 (9)	0.0212 (8)	−0.0068 (7)	−0.0010 (7)	−0.0051 (7)
C19	0.0197 (8)	0.0214 (8)	0.0203 (8)	−0.0046 (7)	−0.0011 (6)	−0.0041 (7)
C20	0.0194 (8)	0.0223 (8)	0.0172 (8)	−0.0057 (7)	−0.0011 (6)	−0.0028 (6)
C21	0.0186 (8)	0.0198 (8)	0.0177 (8)	−0.0026 (6)	−0.0007 (6)	−0.0049 (6)
C22	0.0225 (9)	0.0218 (9)	0.0166 (8)	−0.0053 (7)	−0.0023 (6)	−0.0048 (7)
C23	0.0240 (9)	0.0249 (9)	0.0216 (9)	−0.0075 (7)	−0.0016 (7)	−0.0064 (7)
C24	0.0323 (10)	0.0236 (9)	0.0233 (9)	−0.0080 (8)	−0.0044 (8)	−0.0063 (7)
C25	0.0366 (11)	0.0239 (9)	0.0281 (10)	−0.0018 (8)	−0.0007 (8)	−0.0118 (8)
C26	0.0309 (10)	0.0269 (10)	0.0264 (9)	−0.0033 (8)	0.0051 (8)	−0.0098 (8)
C27	0.0234 (9)	0.0235 (9)	0.0185 (8)	−0.0041 (7)	−0.0010 (7)	−0.0058 (7)
C28	0.0208 (8)	0.0256 (9)	0.0175 (8)	−0.0051 (7)	0.0016 (7)	−0.0041 (7)
C29	0.0395 (12)	0.0253 (10)	0.0417 (12)	−0.0113 (9)	−0.0012 (9)	−0.0120 (9)
O2	0.053 (2)	0.061 (2)	0.064 (3)	−0.009 (2)	−0.025 (2)	−0.027 (2)
C30	0.085 (5)	0.037 (3)	0.084 (5)	−0.008 (3)	0.007 (4)	−0.040 (3)
C31	0.065 (3)	0.046 (3)	0.033 (2)	−0.016 (3)	−0.006 (2)	−0.015 (2)
O2'	0.038 (2)	0.155 (5)	0.034 (2)	−0.015 (3)	−0.0068 (16)	−0.018 (3)
C30'	0.028 (2)	0.053 (3)	0.037 (2)	−0.017 (2)	−0.0039 (19)	−0.004 (2)
C31'	0.034 (3)	0.043 (3)	0.047 (3)	−0.008 (2)	−0.004 (2)	−0.011 (2)

### Geometric parameters (Å, º)

**Table:**

Cl1—C4	1.7371 (19)	C18—C19	1.337 (3)
Cl2—C21	1.7562 (18)	C18—H18	0.9500
O1—C17	1.223 (2)	C19—C20	1.464 (2)
N1—C9	1.318 (2)	C19—H19	0.9500
N1—C1	1.367 (2)	C20—C28	1.381 (3)
N2—C21	1.292 (2)	C20—C21	1.428 (2)
N2—C22	1.375 (2)	C22—C23	1.415 (3)
C1—C2	1.414 (3)	C22—C27	1.419 (3)
C1—C6	1.419 (2)	C23—C24	1.373 (3)
C2—C3	1.369 (3)	C23—H23	0.9500
C2—H2	0.9500	C24—C25	1.420 (3)
C3—C4	1.408 (3)	C24—C29	1.510 (3)
C3—H3	0.9500	C25—C26	1.368 (3)
C4—C5	1.368 (3)	C25—H25	0.9500
C5—C6	1.419 (3)	C26—C27	1.414 (3)
C5—H5	0.9500	C26—H26	0.9500
C6—C7	1.432 (2)	C27—C28	1.411 (3)
C7—C8	1.382 (3)	C28—H28	0.9500
C7—C11	1.487 (2)	C29—H29A	0.9800
C8—C9	1.435 (2)	C29—H29B	0.9800
C8—C17	1.509 (2)	C29—H29C	0.9800
C9—C10	1.506 (3)	O2—C30	1.498 (5)
C10—H10A	0.9800	O2—H2O	0.8400
C10—H10B	0.9800	C30—C31	1.485 (5)
C10—H10C	0.9800	C30—H30A	0.9900
C11—C16	1.397 (2)	C30—H30B	0.9900
C11—C12	1.397 (3)	C31—H31A	0.9800
C12—C13	1.389 (3)	C31—H31B	0.9800
C12—H12	0.9500	C31—H31C	0.9800
C13—C14	1.393 (3)	O2'—C30'	1.358 (4)
C13—H13	0.9500	O2'—H2O'	0.8400
C14—C15	1.382 (3)	C30'—C31'	1.462 (4)
C14—H14	0.9500	C30'—H30C	0.9900
C15—C16	1.388 (3)	C30'—H30D	0.9900
C15—H15	0.9500	C31'—H31D	0.9800
C16—H16	0.9500	C31'—H31E	0.9800
C17—C18	1.478 (2)	C31'—H31F	0.9800
			
C9—N1—C1	118.61 (15)	C18—C19—H19	118.3
C21—N2—C22	117.46 (16)	C20—C19—H19	118.3
N1—C1—C2	117.55 (16)	C28—C20—C21	115.04 (16)
N1—C1—C6	123.01 (16)	C28—C20—C19	122.32 (16)
C2—C1—C6	119.43 (17)	C21—C20—C19	122.63 (16)
C3—C2—C1	121.03 (17)	N2—C21—C20	127.10 (17)
C3—C2—H2	119.5	N2—C21—Cl2	115.16 (13)
C1—C2—H2	119.5	C20—C21—Cl2	117.73 (14)
C2—C3—C4	118.94 (17)	N2—C22—C23	118.58 (16)
C2—C3—H3	120.5	N2—C22—C27	121.35 (17)
C4—C3—H3	120.5	C23—C22—C27	120.07 (17)
C5—C4—C3	122.15 (18)	C24—C23—C22	120.69 (18)
C5—C4—Cl1	119.79 (15)	C24—C23—H23	119.7
C3—C4—Cl1	118.05 (14)	C22—C23—H23	119.7
C4—C5—C6	119.56 (17)	C23—C24—C25	119.05 (18)
C4—C5—H5	120.2	C23—C24—C29	121.56 (19)
C6—C5—H5	120.2	C25—C24—C29	119.39 (18)
C5—C6—C1	118.81 (16)	C26—C25—C24	121.22 (18)
C5—C6—C7	123.40 (16)	C26—C25—H25	119.4
C1—C6—C7	117.79 (16)	C24—C25—H25	119.4
C8—C7—C6	118.00 (16)	C25—C26—C27	120.78 (19)
C8—C7—C11	121.33 (16)	C25—C26—H26	119.6
C6—C7—C11	120.65 (16)	C27—C26—H26	119.6
C7—C8—C9	119.98 (16)	C28—C27—C26	123.67 (18)
C7—C8—C17	121.81 (16)	C28—C27—C22	118.16 (17)
C9—C8—C17	118.21 (16)	C26—C27—C22	118.17 (18)
N1—C9—C8	122.50 (16)	C20—C28—C27	120.84 (17)
N1—C9—C10	115.95 (16)	C20—C28—H28	119.6
C8—C9—C10	121.49 (16)	C27—C28—H28	119.6
C9—C10—H10A	109.5	C24—C29—H29A	109.5
C9—C10—H10B	109.5	C24—C29—H29B	109.5
H10A—C10—H10B	109.5	H29A—C29—H29B	109.5
C9—C10—H10C	109.5	C24—C29—H29C	109.5
H10A—C10—H10C	109.5	H29A—C29—H29C	109.5
H10B—C10—H10C	109.5	H29B—C29—H29C	109.5
C16—C11—C12	118.79 (16)	C31—C30—O2	109.7 (4)
C16—C11—C7	121.46 (16)	C31—C30—H30A	109.7
C12—C11—C7	119.61 (16)	O2—C30—H30A	109.7
C13—C12—C11	120.48 (17)	C31—C30—H30B	109.7
C13—C12—H12	119.8	O2—C30—H30B	109.7
C11—C12—H12	119.8	H30A—C30—H30B	108.2
C12—C13—C14	120.07 (18)	C30—C31—H31A	109.5
C12—C13—H13	120.0	C30—C31—H31B	109.5
C14—C13—H13	120.0	H31A—C31—H31B	109.5
C15—C14—C13	119.75 (18)	C30—C31—H31C	109.5
C15—C14—H14	120.1	H31A—C31—H31C	109.5
C13—C14—H14	120.1	H31B—C31—H31C	109.5
C14—C15—C16	120.35 (17)	C30'—O2'—H2O'	109.5
C14—C15—H15	119.8	O2'—C30'—C31'	123.1 (5)
C16—C15—H15	119.8	O2'—C30'—H30C	106.5
C15—C16—C11	120.55 (17)	C31'—C30'—H30C	106.5
C15—C16—H16	119.7	O2'—C30'—H30D	106.5
C11—C16—H16	119.7	C31'—C30'—H30D	106.5
O1—C17—C18	122.03 (17)	H30C—C30'—H30D	106.5
O1—C17—C8	119.42 (16)	C30'—C31'—H31D	109.5
C18—C17—C8	118.49 (15)	C30'—C31'—H31E	109.5
C19—C18—C17	120.28 (17)	H31D—C31'—H31E	109.5
C19—C18—H18	119.9	C30'—C31'—H31F	109.5
C17—C18—H18	119.9	H31D—C31'—H31F	109.5
C18—C19—C20	123.37 (17)	H31E—C31'—H31F	109.5
			
C9—N1—C1—C2	179.21 (16)	C14—C15—C16—C11	−0.5 (3)
C9—N1—C1—C6	−2.3 (3)	C12—C11—C16—C15	0.9 (3)
N1—C1—C2—C3	178.39 (16)	C7—C11—C16—C15	−174.92 (17)
C6—C1—C2—C3	−0.1 (3)	C7—C8—C17—O1	−125.81 (19)
C1—C2—C3—C4	1.3 (3)	C9—C8—C17—O1	54.8 (2)
C2—C3—C4—C5	−0.3 (3)	C7—C8—C17—C18	56.8 (2)
C2—C3—C4—Cl1	179.79 (14)	C9—C8—C17—C18	−122.50 (18)
C3—C4—C5—C6	−1.9 (3)	O1—C17—C18—C19	−4.1 (3)
Cl1—C4—C5—C6	177.96 (13)	C8—C17—C18—C19	173.13 (17)
C4—C5—C6—C1	3.1 (3)	C17—C18—C19—C20	178.62 (16)
C4—C5—C6—C7	−176.86 (16)	C18—C19—C20—C28	−36.6 (3)
N1—C1—C6—C5	179.47 (16)	C18—C19—C20—C21	144.44 (19)
C2—C1—C6—C5	−2.1 (3)	C22—N2—C21—C20	−1.2 (3)
N1—C1—C6—C7	−0.6 (3)	C22—N2—C21—Cl2	179.98 (12)
C2—C1—C6—C7	177.86 (16)	C28—C20—C21—N2	2.1 (3)
C5—C6—C7—C8	−176.93 (16)	C19—C20—C21—N2	−178.84 (17)
C1—C6—C7—C8	3.1 (2)	C28—C20—C21—Cl2	−179.09 (13)
C5—C6—C7—C11	4.8 (3)	C19—C20—C21—Cl2	−0.1 (2)
C1—C6—C7—C11	−175.13 (15)	C21—N2—C22—C23	179.24 (16)
C6—C7—C8—C9	−2.9 (2)	C21—N2—C22—C27	−0.7 (3)
C11—C7—C8—C9	175.35 (15)	N2—C22—C23—C24	179.13 (16)
C6—C7—C8—C17	177.79 (15)	C27—C22—C23—C24	−0.9 (3)
C11—C7—C8—C17	−4.0 (3)	C22—C23—C24—C25	−0.5 (3)
C1—N1—C9—C8	2.6 (3)	C22—C23—C24—C29	180.00 (17)
C1—N1—C9—C10	179.90 (15)	C23—C24—C25—C26	1.2 (3)
C7—C8—C9—N1	0.0 (3)	C29—C24—C25—C26	−179.23 (19)
C17—C8—C9—N1	179.33 (16)	C24—C25—C26—C27	−0.6 (3)
C7—C8—C9—C10	−177.14 (16)	C25—C26—C27—C28	179.13 (18)
C17—C8—C9—C10	2.2 (2)	C25—C26—C27—C22	−0.8 (3)
C8—C7—C11—C16	54.5 (2)	N2—C22—C27—C28	1.6 (3)
C6—C7—C11—C16	−127.30 (18)	C23—C22—C27—C28	−178.40 (16)
C8—C7—C11—C12	−121.2 (2)	N2—C22—C27—C26	−178.53 (16)
C6—C7—C11—C12	57.0 (2)	C23—C22—C27—C26	1.5 (3)
C16—C11—C12—C13	−0.9 (3)	C21—C20—C28—C27	−1.1 (3)
C7—C11—C12—C13	174.99 (19)	C19—C20—C28—C27	179.88 (16)
C11—C12—C13—C14	0.5 (3)	C26—C27—C28—C20	179.54 (18)
C12—C13—C14—C15	−0.2 (3)	C22—C27—C28—C20	−0.6 (3)
C13—C14—C15—C16	0.2 (3)		

### Hydrogen-bond geometry (Å, º)

**Table:**

*D*—H···*A*	*D*—H	H···*A*	*D*···*A*	*D*—H···*A*
C15—H15···N2^i^	0.95	2.55	3.335 (2)	140
C25—H25···O1^ii^	0.95	2.45	3.394 (3)	170
C26—H26···Cl1^iii^	0.95	2.75	3.654 (2)	159

Symmetry codes: (i) *x*−1, *y*, *z*; (ii) *x*, *y*+1, *z*; (iii) −*x*+1, −*y*+1, −*z*+1.
